# Outcomes of Cranioplasty: A Single-Center Experience

**DOI:** 10.7759/cureus.35213

**Published:** 2023-02-20

**Authors:** Mahmoud A Fallatah, Abdulaziz Aldahlawi, Emad M Babateen, Saif Saif, Waleed Alnejadi, Mouaz Bamsallm, Ahmed Lary

**Affiliations:** 1 Medicine, King Saud Bin Abdulaziz University for Health Sciences College of Medicine, Jeddah, SAU; 2 Neurosurgery, King Abdulaziz Medical City Jeddah, Jeddah, SAU; 3 Neurosurgical Oncology, National Guard Hospital, Jeddah, SAU

**Keywords:** morbidity and mortality, complication, infection, outcome, cranioplasty, craniectomy

## Abstract

Background

Cranioplasty (CP) is a common cranial reconstructive procedure. It is performed after craniectomy due to various causes such as relieving increased intracranial pressure, infection, and tumor infiltration. Although CP is an easy procedure, it is associated with a high rate of complications. We aimed to retrospectively investigate the outcomes of CP at the King Abdulaziz Medical City, Ministry of National Guard Health Affairs, Jeddah (KAMC-J).

Methods

This is a retrospective observational study that included all patients who had CP (first time or redo) at KAMC-J from 1st January 2010 to 31st December 2020. Patients with congenital cranial malformation were excluded.

Result

A total of 68 patients underwent CP. Of those, 23 (34%) had complications. The most common complication was infection (10.3%). Twelve of the 23 patients had major complications that necessitated reoperation. Of those 12, six underwent redo CP; three out of these six patients had further complications which were also managed surgically. On bivariate analysis, cranial defects over 50 cm² were associated with a higher rate of both infection and hydrocephalus (p=0.018) while the frontal site was associated with a higher rate of infection (p=0.014). Moreover, traumatic brain injury as an etiology was exclusively associated with post-cranioplasty hydrocephalus (p=0.03).

Conclusion

Patients undergoing CP after craniectomy are prone to a considerably high rate of adverse outcomes. The overall rate of complications in this study was 34%, with an infection rate of 10.3% and a 1.5% mortality rate. Consistent with other studies, larger cranial defects as well as frontal sites have a higher rate of infection.

## Introduction

Cranioplasty (CP) is a common cranial reconstructive procedure that aims to restore the shape and function of the calvarium. It is usually performed for patients who required a craniectomy due to various causes such as relieving increased intracranial pressure, stroke, trauma, infection, and tumor infiltration [1]. There are two categories of materials that have been used in CP: autografts, and alloplastic grafts; the latter comprises different types of material such as alumina ceramics and methyl methacrylate (MMA). There is still no consensus about the optimal material to be used in CP and the choice depends on multiple factors such as the patient's age, size and location of the defect, preference of the surgeon, and the primary cause of the cranial bone removal [2].

Although CP is a relatively easy-to-perform operation in neurosurgery, it can be associated with a high rate of complications ranging from 12% and reaching up to 50% [3,4]. These complications can also range from minor, which requires only observation, to major which needs reoperation as reported in many studies [5-7]. The complications can be classified as intraoperative or postoperative, however, postoperative complications have been reported more frequently in the literature [8]. The postoperative complications which have been reported frequently are wound infection, bone flap infection, wound dehiscence, intracranial collections, sunken bone flap, bone flap resorption (BFR), and seizure [9]. The rate of each complication and the related risk factor varies considerably in the literature with infection as the most occurring in one study (53.21% out of the total complication rate 10.9), and BFR in another (54.7% out of the total complication rate 36%) [7,10]. In addition, several predictive parameters have been correlated with some of the complications such as the material being used, the location of the skull defect, and the presence of a ventriculoperitoneal (VP) shunt at the time of CP [11-13]. Nevertheless, there is still inconsistency and variation regarding these predictive factors in the literature [14]. Moreover, there is a lack of sufficient studies in Saudi Arabia regarding the outcomes of CP and a need for more data about the current status of CP outcomes in Saudi Arabia.

Therefore, considering the reported morbidity associated with CP and the lack of sufficient data in Saudi Arabia, the aim of our study was to investigate the outcomes of CP and evaluate some factors that might affect the outcomes in King Abdulaziz Medical City, Ministry of National Guard Health Affairs, Jeddah, Saudi Arabia (KAMC-J).

## Materials and methods

This retrospective observational study was conducted on consecutive patients of all age groups who had undergone CP at KAMC-J between a 10-year period (1st January 2010 to 31st December 2020). Patients' identification and data extraction were obtained from the KAMC-J database. Missing information was extracted from the patient charts. Patients with a congenital cranial malformation (e.g., craniosynostosis) and patients whose data was incomplete at the time of data collection were excluded. The study was approved by the Institutional Review Board of King Abdullah International Medical Research Centre (approval no. SP21J/153/04).

Data acquisition

Extracted data included age, gender, medical history, indication for craniectomy, the interval between the craniectomy and the CP, location of CP, size of the cranial defect, type of material used for CP (e.g., autograft, MMA, Titanium mesh, etc.), presence of VP shunt at time of CP, intraoperative complication, postoperative complication, the interval between CP and postoperative complication, management of postoperative complication, first time CP or redo, the reason for a redo, the material used for the redo, and the result of the redo.

Cranial defect size acquisition and autologous bone storage methods

The relative 2D size of the cranial defect was obtained by adapting the method of Dünisch et al. i.e., by using a preoperative sagittal plain skull X-ray or a sagittal CT image, and the following formula: A = p/4 × B ×b, where p is a constant, and B and b are the largest diameters of an elliptic area [15].

The preservation method of autologous bone in our institution is by cryopreservation in a freezer located in the operating room after rinsing the flap with 0.9% saline solution and placing it in a sterile bag.

Statistical analysis

Frequencies and percentages were used to describe qualitative variables, and median (non-normally distributed data) or mean (normally distributed data) with standard deviation for continuous variables. Contingency tables with hypothesis testing were used by analysis of variance. Pearson’s chi-square test or Fisher’s exact test was used to display non-parametric variables and counts of complications. Multivariate logistic regression for risk factor analysis could not be performed due to the small sample size. Any missing data were not included in the analysis. The SPSS software version 23 (IBM Corp., Armonk, NY, USA) was used for analysis. The confidence level was set at 95%, and statistical significance was determined at p<0.05.

## Results

Patient demographics 

Sixty-eight patients who fulfilled the inclusion criteria were included in the study with a median age of 40 years (IQR, 22 to 55). There was a slight male (57.4%) to female (42.6%) predominance. Tumor resection was the most common indication for craniectomy (54.3%) followed by traumatic head injury (THI) at 25%, and infection at 13.2%. Frontal location was the most common site for cranioplasty (19.9%). The median size of the cranial defect was 39 cm ² (IQR, 13 cm ² to 71 cm ²). The median interval between craniectomy and cranioplasty was one day (IQR, 1 to 180 days). Titanium mesh was the most used material (41.2%). Detailed demographics are described in Table 1.

**Table 1:** Patient characteristics and demographics CP: Cranioplasty, HTN: Hypertension, DM: Diabetes mellitus, THI: Traumatic head injury, PMMA: polymethylmethacrylate, CVA: Cerebrovascular accident, VP: Ventriculoperitoneal

Characteristics	Frequency	Percentage
Gender		
Female	29	42.6%
Male	39	57.4%
Age median and IQR=40 (22 to 55) years		
Pediatric <18	15	21%
Adults ≥18	53	78%
Comorbidities		
HTN	5	5.9%
DM	4	7.4%
HTN & DM	7	10.4%
Interval between craniectomy & CP median and IQR=1 (1-180) days		
Location of cranioplasty		
Frontal	20	29.4%
Frontoparietotemporal	6	8.8%
Frontoparietal	7	10.3%
Frontotemporal	6	8.8%
Parietal	5	7.4%
Parietotemporal	5	7.4%
Temporal	6	8.8%
Parietooccipital	1	1.5%
Occipital	12	17.6%
Indications for craniectomy		
THI	17	25%
CVA	4	6%
Tumor	37	54.3%
Infection	9	13.2%
Intra-operative swelling	1	1.5%
Size of CP		
0-49 cm²	40	61.5%
≥50 cm²	25	38.5%
VP shunt during CP		
Absent	58	85.3%
Present	10	14.7%
Material of CP		
Autologous	10	14.7%
PMMA	9	13.2%
Titanium mesh	28	41.2%
PMMA+Titanium mesh	21	30.9%

Complications

The overall complication rate was 33.9% (intraoperative 1.5%, postoperative 32.4%). The only intraoperative complication was severe bleeding that required a blood transfusion for a one-year-old boy. Most postoperative complication was attributed to infection (10.3%) followed by minor complications (8.8%), hydrocephalus (6%), new onset seizure (4.4%), loose bone graft (1.5%), and death (1.5%) as shown in Table 2 and Table 3.

**Table 2:** Postoperative major complications CP: Cranioplasty

Interval between CP and post-op complications (days) Median and IQR=11 (3-86)		
Major Complications	Frequency	Percentage
Death	1	1.5%
Infection	7	10.3%
Hydrocephalus	4	6%
Seizure	3	4.4%
Loose bone graft	1	1.5%

**Table 3:** Postoperative minor complications N/V: Nausea and vomiting, DVT: Deep venous thrombosis, CSF: Cerebrospinal fluid

Minor complications	Frequency
N/V & Headache	1
Angioedema	1
Subgaleal collection	2
Lower extremity DVT	1
CSF leak	1
Total percentage	8.8%

**Figure 1:**
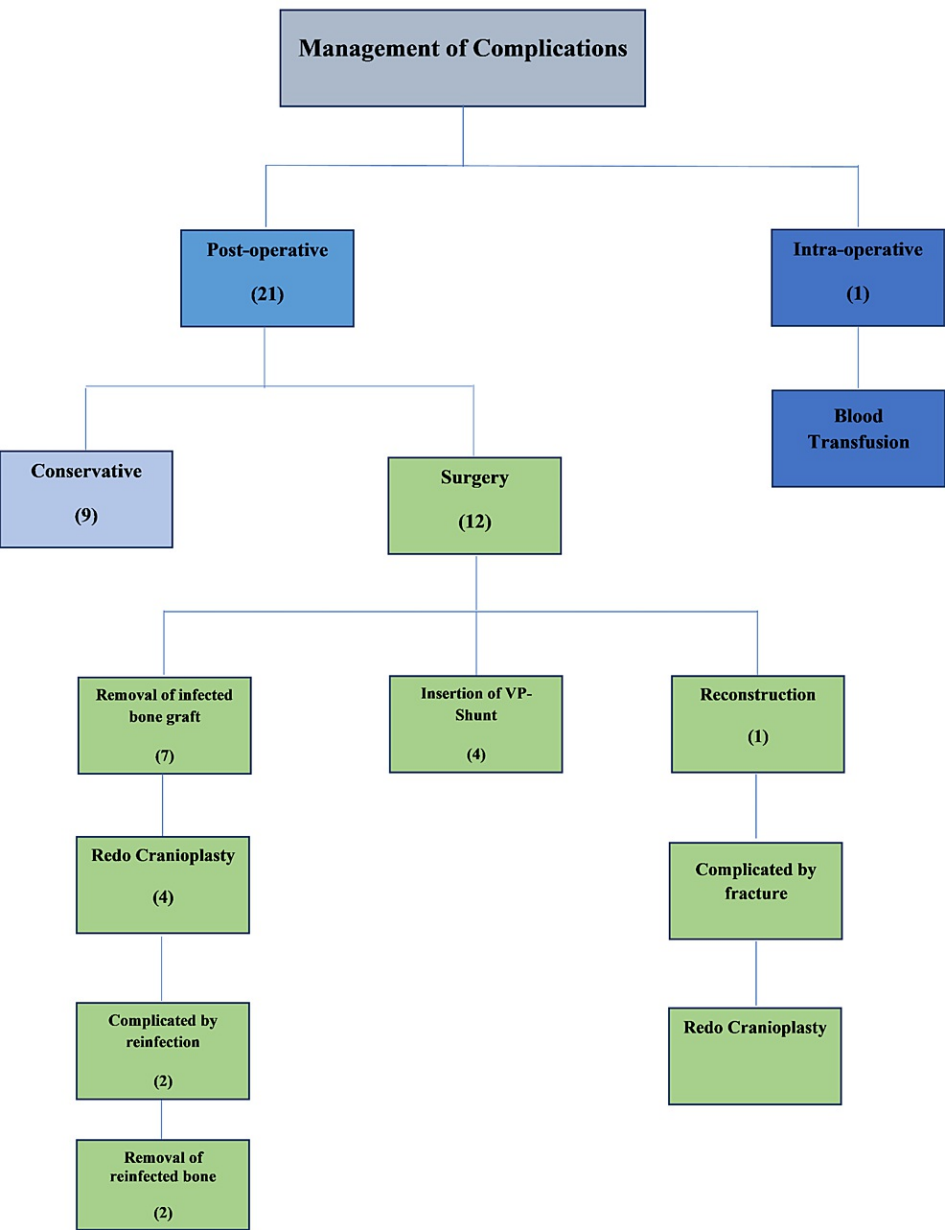
A flow chart showing the course undergone by patients with CP complications VP: Ventriculopertioneal, CP: Cranioplasty

The median Interval between CP and postoperative complication in days was 11 (IQR 3-86). As part of the management of postoperative complications, 12 patients (57.1%) required surgical revision (removal of infected bone graft, insertion of VP shunt, reconstruction), and 9 (42.9%) were treated conservatively (Figure 1). 

Infection

Seven patients sustained post-CP infection. A positive culture for methicillin-resistant *Staphylococcus Aureus* (MRSA) was seen in three patients, *Acinobacter Baumanii* in one; *Pseudomonas Aeruginosa* in one; multidrug resistance (MDR) *Klebsiella Pneumoniae* in one; and two concurrent organisms i.e., *Citrobacter Koseri, *and *Klebsiella Pneumoniae *in one patient. Interestingly, the patient with the isolated *K. Pneumoniae *was a victim of THI and had a craniectomy outside our hospital. His bone flap was preserved in his abdomen and then was transferred to our hospital. He was found to have intra-axial and extra-axial abscesses as well as in his abdomen, and both sites were positive for*K. Pneumoniae.* All patients underwent surgical removal of the bone flap.

Death

The one fatality in our study occurred in a pediatric patient. He underwent a craniectomy due to tumor infiltration. After five months, he presented with tumor recurrence and the tumor was infiltrating the skin through the bony defect. The surgeon performed tumor debulking and decided to do CP at the same setting to prevent possible tumor recurrence from infiltrating the skin. A few hours after the operation, the patient progressively deteriorated and was found to have massive brain swelling after cranioplasty (MBSC). He returned to the operating table and underwent emergent decompressive craniectomy (DC). Unfortunately, he did not survive long after.

Reoperations

Six patients (8.8%) had redo CP; four out of these six patients had the redo after the removal of the infected graft and systemic clearance of the infection. The fifth was not due to complication but rather due to tumor recurrence. And the last one after the original CP was complicated by a loose graft. Moreover, three out of the six patients who had redo CP suffered from further failure; two of them were from the four patients who had the redo because of graft infection. Their redo was complicated by graft reinfection and they underwent surgical removal of the graft without further CP. And the third one was the one with a loose graft after suffering a crack fracture of the graft, and underwent redo CP for the third time (Figure 1).

Statistical analysis

We divided the postoperative complication into two groups: minor and major, according to the degree of effect on the patients whether they required invasive intervention or not. The minor group included those who developed nausea/vomiting, headache, angioedema, subgaleal collection, lower extremity deep vein thrombosis (DVT), and cerebrospinal fluid (CSF) leak. On the other hand, the major group included death, infection, hydrocephalus, seizure, and loose bone graft. Only the major group was included in the analysis. We tried to find risk associated with major postoperative complications and with the specific type of complication. We found that cranial defects over 50 cm² were associated with a higher rate of infection and hydrocephalus (p=0.018) while the frontal site was only associated with a higher rate of infection (p=0.014). Moreover, THI as an etiology was exclusively associated with post-cranioplasty hydrocephalus (p=0.03). Conversely, the specific type of complications was not significantly influenced by age group, gender, comorbidities, the time interval between craniectomy and CP, the material used for CP, and the presence of VP shunt as shown in Table 4.

**Table 4:** Risk associated with the type of postoperative complication (n=16) CP: Cranioplasty, HTN: Hypertension, DM: Diabetes mellites, CVA: Cerebrovascular accident, VP: Ventriculoperitoneal, PMMA: Polymethylmethacrylate, CVA: Cerebrovascular accident

Variables	Death	Infection	Hydrocephalus	Seizure	Loose graft	Test statistic, p-value
Gender						
Female	0(0)	5(71.4)	0(0)	2(28.6)	0(0)	Fisher’s exact=7.00, (p=0.07)
Male	1(11.1)	2(22.2)	4(44.4)	1(11.1)	1(11.1)	
Age						
Pediatric	1(33.3)	1(33.3)	0(0)	0(0)	1(33.3)	Fisher’s exact=7.30, (p=0.07)
Adults	0(0)	6(46.2)	4(30.8)	3(23.1)	1(6.3)	
Comorbidities						
HTN	1(8.3)	5(41.7)	4(33.3)	1(8.3)	1(8.3)	Fisher’s exact=4.41, (p=0.36)
DM	0(0)	2(50)	0(0)	2(50)	0(0)	
HTN & DM	0(0)	0(0)	0(0)	0(0)	0(0)	
Interval between craniectomy & CP						
>3 M	0(0)	5(62.5)	2(25)	1(12.5)	0(0)	Fisher’s exact=3.60, (p=0.45)
<3 M	1(12.5)	2(25)	2(25)	2(25)	1(12.5)	
Location of CP						
Frontal	0(0)	4(100)	0(0)	0(0)	0(0)	Fisher’s exact=30.54, (p=0.014)
Frontoparietotemporal	0(0)	1(33.3)	1(33.3)	0(0)	1(33.3)	
Frontoparietal	0(0)	0(0)	0(0)	2(100)	0(0)	
Frontotemporal	1(50)	0(0)	0(0)	1(50)	0(0)	
Parietotemporal	0(0)	1(50)	1(50)	0(0)	0(0)	
Temporal	0(0)	0(0)	2(100)	0(0)	0(0)	
Parietooccipital	0(0)	1(100)	0(0)	0(0)	0(0)	
Indications for craniectomy						
THI	0(0)	1(16.7)	4(66.7)	0(0)	1(16.7)	Fisher’s exact=17.55, (p=0.03)
CVA	0(0)	1(100)	0(0)	0(0)	0(0)	
Tumor	0(0)	4(80)	0(0)	1(20)	0(0)	
Infection	1(25)	1(25)	0(0)	2(50)	0(0)	
Size of CP						
0-49 cm	0(0)	0(0)	0(0)	2(66.7)	1(33.3)	Fisher’s exact=9.00, (p=0.018)
≥50 cm²	1(7.7)	7(53.8)	4(30.8)	1(7.7)	0(0)	
VP shunt during CP						
Absent	1(7.1)	6(42.9)	4(28.6)	2(14.3)	1(7.1)	Fisher’s exact=3.33, (p=0.80)
Present	0(0)	1(50)	0(0)	1(50)	0(0)	
Material of CP						
Autologous	0(0)	1(20)	4(80)	0(0)	0(0)	Fisher’s exact=15.80, (p=0.43)
PMMA	1(25)	2(50)	0(0)	0(0)	1(25)	
Titanium mesh	0(0)	2(66.7)	0(0)	1(33.3)	0(0)	
PMMA+Titanium mesh	0(0)	2(50)	0(0)	2(50)	0(0)	

## Discussion

Cranioplasty is a substantial secondary procedure that is necessitated by cosmetic reasons and a protective function for the brain [1,2]. Many studies also suggested contributory factors of CP in improving postoperative neurological status, namely cognitive and motor function, via restoring cerebrovascular and CSF hydrodynamics [16,17]. Despite being a routine and straightforward procedure, a high rate of procedural complications has been recognized in the last decade. In our present study, we investigated the outcome of CP in a single tertiary center. We identified the overall complication rate to be 33.9% and a mortality rate of 1.5%, which is similar to several published studies [5,18,19].

Infection

In line with multiple studies with comparable sample sizes, post-CP infection is the most common attributable factor for postoperative complications [5,10]. Many studies suggested several factors influencing this complication. For instance, frontal site, and violation of the frontal air sinus as well were demonstrated as definitive risk factors of post-CP infection [20,21]. However, although the frontal site was significantly associated with post-CP infection in our study (p=0.014), we found only one case of violated frontal air sinus sustained post-CP infection. Nevertheless, this one case sustained reinfection to the redo CP, which may indicate the difficult and devastating outcome seen in this subgroup of patients [20,21,22].

Another studied factor was the timing of CP after DC, a study found that early CP (defined as <12 weeks) was associated with a lower infection rate than the late group (defined as ≥12 weeks) despite not reaching statistical significance [23]. Nonetheless, several other studies found the interval timing did not affect post-CP infection [3,19,24,25], including a recent large meta-analysis conducted by Malcom et al., where they found no difference between early CP group (defined as less than 90 days) and late CP group (defined as more than 90 days) [26]. In our study, it is hard to draw any conclusion regarding the impact of timing on post-CP infection since most of the indications for craniectomy were due to tumors that needed no delay in performing the CP.

We also found that the type of material used in CP, and the presence of VP shunt did not affect post-CP infection. The former is consistent with several studies and suggests that the material used in CP is unlikely to influence the post-CP infection rate [4,7,25,27]. Conversely, the presence of VP shunt during CP was found to pose a significant risk for post-CP infection in a study by Tsang et al., and overall complication rate as well in other studies [6,28,29].

Cranial defect over 50 cm² was significantly associated with post-CP infection in our study (p=0.018). Along with previously described studies, Alkhaibaryet al. also found larger cranial defects were a predictor of post-CP infection [30]; possibly due to longer operating time, or due to an increased skin tension that may lead to wound dehiscence [31]. Nevertheless, one needs to consider the impact of the patient’s condition on wound healing, specifically in the immunocompromised state seen in the subgroup of patients with tumors (which constituted most of the infected cases in our study) that could be attributable to the malignancy condition itself, use of cortisone, chemotherapy treatment or exposure to radiation and poor nutritional state [31].

Hydrocephalus

Shunt-dependent hydrocephalus is a well-observed phenomenon post CP, and it is seen frequently in patients exposed to decompressive hemicraniectomy. Although it is well recognized, the exact mechanism behind it is still unknown, and it is unclear if the hydrocephalus is attributable to the severity of the primary insult, the craniectomy, or the CP itself [26]. This unclarity is partly due to various findings that line with one of the possible causes. For instance, it is well-established that subarachnoid hemorrhage (SAH) secondary to THI and or hemorrhagic stroke impacts the CSF dynamics through flow obstruction caused by clot formation and blood products that compromise the subarachnoid granulations, as well as by formation of adhesions in the ventricles and cisterns that lead to chronic hydrocephalus. This implies that the primary insult influences post-CP hydrocephalus [32-34].

On the other hand, a growing body of evidence showed that DC is an independent risk factor for developing post-traumatic hydrocephalus (PTH) regardless of CP, including a recent prospective study by Goldschmidt et al. [35-37]. Recently, a proposed physiological mechanism that may play a role in CSF homeostasis has been suggested as an explanation for the risk of DC on PTH. For instance, because the arachnoid granulations function as pressure-dependent unidirectional valves due to their morphology, it requires pulsatile pressure and the CSF absorption is maintained under pulsatile pressure changes [38,39]. Hence, large DC leads to PTH via impairment of the essential pulsatile intracranial CSF dynamics when the cranial vault is transformed from a closed system to an open box [40,41]. Waziri et al. supported this notion by their observation of flattening the normal intracranial pressure waveform during intraparenchymal pressure monitoring after DC [40].

In addition, this concept is also used to explain how early CP may prevent the development of PTH; particularly by restoring the balance of normal CSF pressures before prolonged pressure disturbance leads to permanent dysfunction of arachnoid granulation resulting in the appearance of PTH in late CP. Again, this is supported by several studies that found a significantly lower rate of PTH in early CP (within two months) compared to the late group [40-42].

However, Honeybul et al. found no effect of the timing of CP on PTH and suggested instead the severity of primary brain injury as the cause [37]. Moreover, contrary findings have also been reported by Morton et al., in which a lower rate of PTH was seen in CP performed later than three months [43]. Furthermore, Ozoner et al. assessed the possible effects of the CP and duraplasty materials on PTH development as it was postulated that it may increase the probability of arachnoid adhesions, but no association between CP/duraplasty materials and PTH development was found [41].

Several other risk factors have also been reported to be a predictor of PTH such as the presence of SAH, younger age, and low Glasgow coma scale (GCS) [44]. In the current study, all patients who developed hydrocephalus were those with THI as an initial injury with subsequent decompressive hemicraniectomy (p=0.03). And all had early CP within two months, and eventually all required permeant placement of VP shunt. Moreover, 3/4 were over 40 years old, and only one patient was 38 years old. Our findings, thus, contradict the previously described risk association between early CP and younger age with hydrocephalus development. However, the risk of the presence of SAH and the severity of the primary brain injury remain as possible causes of PTH in our study. Nevertheless, this is to be determined via a larger sample size and prospective studies.

Seizure

New onset seizure is another frequently reported complication post CP, with an incidence rate between 2.7% to 30.3% [45]. Several risk factors have been identified as contributory to its incidence including the time interval between craniectomy and CP, patient age at CP, presence of VP shunt at time of CP, type of material used, the indication for the craniectomy, hypertension (HTN), and diabetes mellitus (DM) [46]. However, these risk factors once again are inconsistent in the literature, and some even are contradictory. It is also difficult to determine whether the incidence of post-CP seizure is only because of the initial disease that led to the craniectomy, or if it is a complication of the CP itself. Many authors supported the former idea. 

In addition, despite the exact underlying pathogenesis being unknown, different mechanisms have been proposed to explain the pathogenesis behind the occurrence of new-onset seizure post CP based on the initial disease through a common understanding of epileptogenesis. For instance, the destruction of cell membranes and increased release of glutamate along with disturbance of electrolyte balance and secretion of free fatty acids are thought to be essential in sufferers of post-ischemic seizures [47]. On the other hand, in cases of traumatic brain injury, neuroinflammation, tauopathy, and mediation by toll-like receptors are believed to cause seizures in 10% to 20% of patients [48], while the byproduct of blood metabolism is thought to be the crucial cause for those who sustained intracranial hemorrhages (ICH) [47].

In contrast, other different mechanisms in favor of the direct consequence of the CP procedure itself upon developing post-CP seizure also have been proposed. Walcott et al. for example suggested that some instances of minimal brain tissue manipulation during the dissection of an extradural plane to facilitate the smooth contour of the bony cranial construct are common in CP, which may precipitate seizure activity in already susceptible brain tissue [18]. Similarly, Wang et al. proposed that the post-traumatic contusion healing process and or exposure of the temporalis muscle during CP may cause scar formation that leads to an abnormal electrical discharge across the scar area inducing seizures [49]. In addition, they also found that autologous fascial repair was an independent risk factor for post-CP seizure, and they observed revascularization between autologous fascia and the cerebral cortex after DC that resulted in traction with a subsequent cortical insult that might induce early seizures in CP patients [49].

However, recently, Hirschmann et al. argued that new-onset seizures post CP are independent of the procedure of CP, and are more likely to be caused by the initial disease [46]. They supported this claim with the findings of their study where they observed that only the time of follow-up was significantly associated with the incidence of new-onset seizures post CP [46]. Moreover, based on their argument they suggested the following explanation: timing of CP only resembles a point in time according to which seizures will be classified and determined to be either pre-existing or new-onset seizure post CP. Therefore, patients undergoing late CP will be more likely to be classified in the pre-existing group and excluded from the study, while on the other hand, more patients with early CP will be included in the study [46]. This explanation is based on their findings that the median time of follow-up in patients with new onset post-CP seizure was three times longer than of patients without a new-onset post-CP seizure in addition to the observation that most cohorts reported insufficient time of follow-up, which led to a considerable proportion of late-onset seizures that will not be observed [46].

In the current study, new onset seizures were found in three patients (4.4%). No specific risk factors were found to be contributory to its incidence. However, interestingly, all patients had their seizures within 24 hours post CP. Which, contrary to Hirschmann et al.'s argument [46], suggests that the CP procedure itself may play some role in provoking seizures even in some indirect way, and not entirely caused by the initial disease. Moreover, in contrast to several studies where new onset seizures were found in patients with THI, hemorrhagic or ischemic strokes, our study noted that the surgical site osteomyelitis infection on the previous craniotomy site was the indication for craniectomy in two patients, and the tumor was the indication in the third. Both of these conditions could precipitate a susceptible brain cortex to be provoked by the subsequent CP procedure.

Death

Death due to CP is rarely seen as a complication of CP. Hence, it wasn’t given much attention in most series that evaluated CP complications. The mortality rate of CP in the literature ranged from 0.7% to 3.16% [50,51]. Most of the series that reported a mortality incidence did not mention the cause. Goedemans et al. reported mortality secondary to a massive postoperative epidural hemorrhage [50]. In addition, a recent systematic review conducted by Robles (&) Cuevas-Solórzano investigated case reports and small case series of post-CP death secondary to MBSC [52]. They identified 19 articles featuring 26 patients with MBSC, and the observed mortality rate was 88%; they concluded that MBSC is a rare but highly lethal complication and possibly underreported condition, triggered by bone flap implantation which causes a sudden increase in intracranial pressure on a brain chronically exposed to intracranial hypotension [52].

Although the cause of the MBSC in our case seemed more likely related to the tumor debulking rather than the CP procedure itself, it is hard to undermine the role of CP in accelerating the unfortunate outcome. 

Reoperations

In our series, we observed that reoperations following CP were extremely high. It is important therefore to emphasize that these reoperations have a heavy burden on the patient's overall condition as well as their affective health because they are predisposed to repeated operations and a long hospital stay. The implementation of strict preventive measures as well as the adherence to a well-developed protocol should be considered to minimize such preventable complications.

Study limitations

Being a retrospective review of a small-sized single-center, this study has naturally inherent deficiencies including recall and observer bias, selection bias, missing patient information, and short follow-up. Moreover, patients who had post-CP infection required reoperation, and this may imply that patients who had a post-CP infection and were managed conservatively may have not been reported, and therefore were not included. This could lead to a less accurate estimation of the true complication rate. 

Unfortunately, performing a detailed functional outcome analysis was not feasible with our small sample size. However, we believe our study will add to the literature and help direct future studies.

## Conclusions

Patients undergoing CP after craniectomy are prone to a considerably high rate of adverse outcomes. These adverse outcomes sometimes can be devastating and require multiple reoperations. The overall rate of complications in this study was 34%, with an infection rate of 10.3% and a mortality rate of 1.5%. Consistent with other studies, larger cranial defects as well as frontal sites have a higher rate of infection. However, in our study, this could not be explained by violation of the frontal air sinus. Furthermore, we found that the post-CP infection rate was not influenced by the time interval between craniectomy and CP, the material used for CP, and the presence of a VP shunt. Our data are too limited to make definitive conclusions, and studies with a larger population and prospective studies are needed to further evaluate CP outcomes.

## References

[1] Dujovny M, Aviles A, Agner C, Fernandez P, Charbel FT (1997). Cranioplasty: cosmetic or therapeutic?. Surg Neurol.

[2] Alkhaibary A, Alharbi A, Alnefaie N, Oqalaa Almubarak A, Aloraidi A, Khairy S (2020). Cranioplasty: a comprehensive review of the history, materials, surgical aspects, and complications. World Neurosurg.

[3] Beauchamp KM, Kashuk J, Moore EE (2010). Cranioplasty after postinjury decompressive craniectomy: is timing of the essence?. J Trauma.

[4] Moreira-Gonzalez A, Jackson IT, Miyawaki T, Barakat K, DiNick V (2003). Clinical outcome in cranioplasty: critical review in long-term follow-up. J Craniofac Surg.

[5] Gooch MR, Gin GE, Kenning TJ, German JW (2009). Complications of cranioplasty following decompressive craniectomy: analysis of 62 cases. Neurosurg Focus.

[6] Tsang AC, Hui VK, Lui WM, Leung GK (2015). Complications of post-craniectomy cranioplasty: risk factor analysis and implications for treatment planning. J Clin Neurosci.

[7] Klinger DR, Madden C, Beshay J, White J, Gambrell K, Rickert K (2014). Autologous and acrylic cranioplasty: a review of 10 years and 258 cases. World Neurosurg.

[8] Sahoo NK, Tomar K, Thakral A, Rangan NM (2018). Complications of ranioplasty. J Craniofac Surg.

[9] Kim JS, Cheong JH, Ryu JI, Kim JM, Kim CH (2015). Bone flap resorption following cranioplasty after decompressive craniectomy: preliminary report. Korean J Neurotrauma.

[10] Brommeland T, Rydning PN, Pripp AH, Helseth E (2015). Cranioplasty complications and risk factors associated with bone flap resorption. Scand J Trauma Resusc Emerg Med.

[11] van de Vijfeijken SE, Münker TJ, Spijker R, Karssemakers LH, Vandertop WP, Becking AG, Ubbink DT (2018). Autologous bone is inferior to alloplastic cranioplasties: safety of autograft and allograft materials for cranioplasties, a systematic review. World Neurosurg.

[12] De Bonis P, Frassanito P, Mangiola A, Nucci CG, Anile C, Pompucci A (2012). Cranial repair: how complicated is filling a "hole"?. J Neurotrauma.

[13] Hirschmann D, Kranawetter B, Kirchschlager C (2021). Cranioplasty following ventriculoperitoneal shunting: lessons learned. Acta Neurochir (Wien).

[14] Iaccarino C, Kolias A, Adelson PD (2021). Consensus statement from the international consensus meeting on post-traumatic cranioplasty. Acta Neurochir (Wien).

[15] Dünisch P, Walter J, Sakr Y, Kalff R, Waschke A, Ewald C (2013). Risk factors of aseptic bone resorption: a study after autologous bone flap reinsertion due to decompressive craniotomy. J Neurosurg.

[16] Honeybul S, Janzen C, Kruger K, Ho KM (2013). The impact of cranioplasty on neurological function. Br J Neurosurg.

[17] Jelcic N, De Pellegrin S, Cecchin D, Della Puppa A, Cagnin A (2013). Cognitive improvement after cranioplasty: a possible volume transmission-related effect. Acta Neurochir (Wien).

[18] Walcott BP, Kwon CS, Sheth SA (2013). Predictors of cranioplasty complications in stroke and trauma patients. J Neurosurg.

[19] Shiban E, Lange N, Hauser A, Jörger AK, Wagner A, Meyer B, Lehmberg J (2020). Cranioplasty following decompressive craniectomy: minor surgical complexity but still high periprocedural complication rates. Neurosurg Rev.

[20] Blum KS, Schneider SJ, Rosenthal AD (1997). Methyl methacrylate cranioplasty in children: long-term results. Pediatr Neurosurg.

[21] Marchac D, Greensmith A (2008). Long-term experience with methylmethacrylate cranioplasty in craniofacial surgery. J Plast Reconstr Aesthet Surg.

[22] De Cola MC, Corallo F, Pria D, Lo Buono V, Calabrò RS (2018). Timing for cranioplasty to improve neurological outcome: a systematic review. Brain Behav.

[23] Piedra MP, Nemecek AN, Ragel BT (2014). Timing of cranioplasty after decompressive craniectomy for trauma. Surg Neurol Int.

[24] Archavlis E, Carvi Y Nievas M (2012). The impact of timing of cranioplasty in patients with large cranial defects after decompressive hemicraniectomy. Acta Neurochir (Wien).

[25] Yadla S, Campbell PG, Chitale R, Maltenfort MG, Jabbour P, Sharan AD (2011). Effect of early surgery, material, and method of flap preservation on cranioplasty infections: a systematic review. Neurosurgery.

[26] Malcolm JG, Rindler RS, Chu JK, Grossberg JA, Pradilla G, Ahmad FU (2016). Complications following cranioplasty and relationship to timing: a systematic review and meta-analysis. J Clin Neurosci.

[27] Chang V, Hartzfeld P, Langlois M, Mahmood A, Seyfried D (2010). Outcomes of cranial repair after craniectomy. J Neurosurg.

[28] Piedra MP, Ragel BT, Dogan A, Coppa ND, Delashaw JB (2013). Timing of cranioplasty after decompressive craniectomy for ischemic or hemorrhagic stroke. J Neurosurg.

[29] Heo J, Park SQ, Cho SJ, Chang JC, Park HK (2014). Evaluation of simultaneous cranioplasty and ventriculoperitoneal shunt procedures. J Neurosurg.

[30] Alkhaibary A, Alharbi A, Abbas M (2020). Predictors of surgical site infection in autologous cranioplasty: a retrospective analysis of subcutaneously preserved bone flaps in abdominal pockets. World Neurosurg.

[31] Riordan MA, Simpson VM, Hall WA (2016). Analysis of factors contributing to infections after cranioplasty: a single-institution retrospective chart review. World Neurosurg.

[32] Dóczi T, Nemessányi Z, Szegváry Z, Huszka E (1983). Disturbances of cerebrospinal fluid circulation during the acute stage of subarachnoid hemorrhage. Neurosurgery.

[33] Hasan D, Tanghe HL (1992). Distribution of cisternal blood in patients with acute hydrocephalus after subarachnoid hemorrhage. Ann Neurol.

[34] Shah AH, Komotar RJ (2013). Pathophysiology of acute hydrocephalus after subarachnoid hemorrhage. World Neurosurg.

[35] Kurland DB, Khaladj-Ghom A, Stokum JA (2015). Complications associated with decompressive craniectomy: a systematic review. Neurocrit Care.

[36] Honeybul S, Ho KM (2012). Incidence and risk factors for post-traumatic hydrocephalus following decompressive craniectomy for intractable intracranial hypertension and evacuation of mass lesions. J Neurotrauma.

[37] Goldschmidt E, Deng H, Puccio AM, Okonkwo DO (2020). Post-traumatic hydrocephalus following decompressive hemicraniectomy: Incidence and risk factors in a prospective cohort of severe TBI patients. J Clin Neurosci.

[38] Bulat M, Klarica M (2011). Recent insights into a new hydrodynamics of the cerebrospinal fluid. Brain Res Rev.

[39] Brinker T, Stopa E, Morrison J, Klinge P (2014). A new look at cerebrospinal fluid circulation. Fluids Barriers CNS.

[40] Waziri A, Fusco D, Mayer SA, McKhann GM 2nd, Connolly ES Jr (2007). Postoperative hydrocephalus in patients undergoing decompressive hemicraniectomy for ischemic or hemorrhagic stroke. Neurosurgery.

[41] Ozoner B, Kilic M, Aydin L, Aydin S, Arslan YK, Musluman AM, Yilmaz A (2020). Early cranioplasty associated with a lower rate of post-traumatic hydrocephalus after decompressive craniectomy for traumatic brain injury. Eur J Trauma Emerg Surg.

[42] Nasi D, Gladi M, Di Rienzo A, di Somma L, Moriconi E, Iacoangeli M, Dobran M (2018). Risk factors for post-traumatic hydrocephalus following decompressive craniectomy. Acta Neurochir (Wien).

[43] Morton RP, Abecassis IJ, Hanson JF (2018). Timing of cranioplasty: a 10.75-year single-center analysis of 754 patients. J Neurosurg.

[44] Fattahian R, Bagheri SR, Sadeghi M (2018). Development of posttraumatic hydrocephalus requiring ventriculoperitoneal shunt after decompressive craniectomy for traumatic brain injury: a systematic review and meta-analysis of retrospective studies. Med Arch.

[45] Shih FY, Lin CC, Wang HC (2019). Risk factors for seizures after cranioplasty. Seizure.

[46] Hirschmann D, Kranawetter B, Tomschik M (2021). New-onset seizures after cranioplasty-a different view on a putatively frequently observed phenomenon. Acta Neurochir (Wien).

[47] Zhao Y, Li X, Zhang K, Tong T, Cui R (2018). The progress of epilepsy after stroke. Curr Neuropharmacol.

[48] Lucke-Wold BP, Nguyen L, Turner RC (2015). Traumatic brain injury and epilepsy: underlying mechanisms leading to seizure. Seizure.

[49] Wang H, Zhang K, Cao H (2017). Seizure after cranioplasty: incidence and risk factors. J Craniofac Surg.

[50] Goedemans T, Verbaan D, van der Veer O (2020). Complications in cranioplasty after decompressive craniectomy: timing of the intervention. J Neurol.

[51] Zanaty M, Chalouhi N, Starke RM (2015). Complications following cranioplasty: incidence and predictors in 348 cases. J Neurosurg.

[52] Robles LA, Cuevas-Solórzano A (2018). Massive brain swelling and death after cranioplasty: a systematic review. World Neurosurg.

